# Recent Trends in Decellularized Extracellular Matrix Bioinks for 3D Printing: An Updated Review

**DOI:** 10.3390/ijms20184628

**Published:** 2019-09-18

**Authors:** Kevin Dzobo, Keolebogile Shirley Caroline M. Motaung, Adetola Adesida

**Affiliations:** 1 International Centre for Genetic Engineering and Biotechnology (ICGEB), Cape Town Component, Wernher and Beit Building (South), UCT Medical Campus, Anzio Road, Observatory, Cape Town 7925, South Africa; 2 Division of Medical Biochemistry and Institute of Infectious Disease and Molecular Medicine, Faculty of Health Sciences, University of Cape Town, Anzio Road, Observatory, Cape Town 7925, South Africa; 3 Department of Biomedical Sciences, Faculty of Science, Tshwane University of Technology, Pretoria 30655, South Africa; smotaung@gmail.com; 4 Department of Surgery, Faculty of Medicine and Dentistry, Li Ka Shing Centre for Health Research Innovation, University of Alberta, Edmonton, AB T6G 2E1, Canada; adesida@ualberta.ca

**Keywords:** regenerative medicine, tissue engineering, decellularized extracellular matrix, 3D bioprinting, bioink, scaffolds, biofabrication, transplantation

## Abstract

The promise of regenerative medicine and tissue engineering is founded on the ability to regenerate diseased or damaged tissues and organs into functional tissues and organs or the creation of new tissues and organs altogether. In theory, damaged and diseased tissues and organs can be regenerated or created using different configurations and combinations of extracellular matrix (ECM), cells, and inductive biomolecules. Regenerative medicine and tissue engineering can allow the improvement of patients’ quality of life through availing novel treatment options. The coupling of regenerative medicine and tissue engineering with 3D printing, big data, and computational algorithms is revolutionizing the treatment of patients in a huge way. 3D bioprinting allows the proper placement of cells and ECMs, allowing the recapitulation of native microenvironments of tissues and organs. 3D bioprinting utilizes different bioinks made up of different formulations of ECM/biomaterials, biomolecules, and even cells. The choice of the bioink used during 3D bioprinting is very important as properties such as printability, compatibility, and physical strength influence the final construct printed. The extracellular matrix (ECM) provides both physical and mechanical microenvironment needed by cells to survive and proliferate. Decellularized ECM bioink contains biochemical cues from the original native ECM and also the right proportions of ECM proteins. Different techniques and characterization methods are used to derive bioinks from several tissues and organs and to evaluate their quality. This review discusses the uses of decellularized ECM bioinks and argues that they represent the most biomimetic bioinks available. In addition, we briefly discuss some polymer-based bioinks utilized in 3D bioprinting.

## 1. Introduction

Regenerative medicine and tissue engineering have shown the ability to influence and impact patients’ treatment [1,2]. Initially started as strategies to try to restore or improve tissues and organ functions, regenerative medicine and tissue engineering are now applied in other areas of research such as drug screening and basic science [3]. Indeed, the last decade has seen an increase in the applications of regenerative medicine knowledge, with several high impact discoveries being published [4,5]. Whilst current treatment options have improved patients’ lives, for example, the inability to regain function of lost, damaged, and diseased tissues and organs has led scientists and clinicians to look elsewhere for solutions. Regenerative medicine and tissue engineering have offered opportunities to solve this problem. There is a need for balance between structural recovery of the tissue or organ versus functional recovery. It might be argued that functional recovery of the lost, diseased or damaged tissue or organ is more important than structural recovery. The development of new biomaterials and bioinks seeks to achieve both goals. However, currently, duplication of structure has been a major challenge with vascularization or its lack thereof being a major contributor. Therefore, the focus of this review is on 3D bioprinting scaffolds that help restore function over time by degrading and stimulating tissue ingrowth in vivo. One limiting factor for regenerative medicine and tissue engineering applications has been the slow approval of regenerative medicine and tissue engineering therapies and constructs [6,7]. Thus, though the investment in regenerative medicine and tissue engineering has been incredible, the returns in terms of safe and reliable therapies and constructs have been slow. It can be argued that the slow pace of approval of therapies and constructs is good for the sake of patients’ safety.

**Figure 1:**
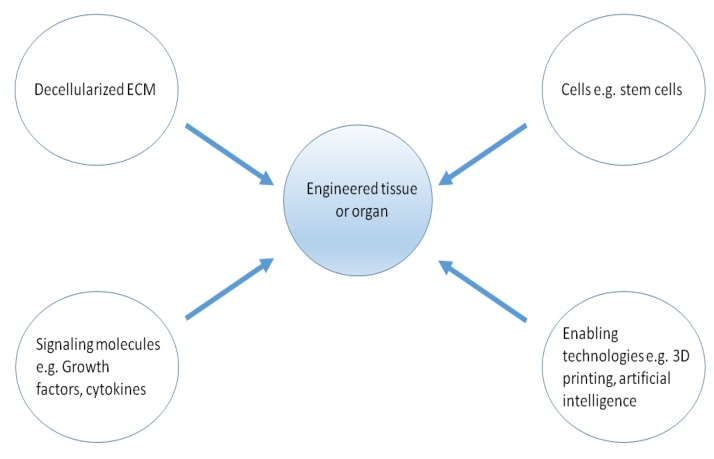
Requirements for engineering of tissues and organs. Decellularized extracellular matrix (dECM), cells, and signaling molecules are mixed in bioreactors in order to initiate tissue or organ formation.

Regenerative medicine and tissue engineering are two words used with appreciable overlap and are used as synonyms in this review (see Appendix A for definitions). Regenerative medicine is a field involved in the restoration of diseased or damaged tissues and organs or the creation of new tissue and organs altogether. Natural biomaterials including collagen, laminin, and fibrin have been used in regenerative medicine applications. In a bid to provide an environment that recapitulates the in vivo microenvironments, biomaterials have been mixed with biochemicals such as growth factors and cytokines. Growth factors and cytokines provide biochemical cues to induce among other processes differentiation of cells or ECM production. However, one of the most promising strategy in terms of recapitulating the target tissue or organ is the use of decellularized ECM (dECM) of the targeted tissue or organ. dECM has the right structure and inductive cues to drive cellular growth and differentiation if stem cells are used (Figure 1) [8]. Currently allogeneic and xenogeneic dECMs have been used for various purposes [8,9]. Several groups use cadavers and donated human parts as a source of dECMs for graft development [9,10,11,12]. Challenges might include the prevention of transmission of infections and diseases, though there have been no reports of retrovirus transmission. There is also a need to avoid an immune reaction to the presence of the new graft or dECM.

Bioinks are required for 3D bioprinting technologies and can also be used during biofabrication. The bringing together of cells, bioactive factors such as growth factors, cellular aggregates, and biomaterials, either via bioprinting or bioassembly, is referred to as biofabrication. Bioinks refer to the mixture of materials and cells used during biofabrication and bioprinting (see Appendix A for Definitions). Different cells and biomaterials can be bioprinted into products for regenerative medicine and tissue engineering. Bioprinting decellularized tissue has one major advantage of the ability to control the position and placement of cells and biomaterials to produce scaffolds/constructs or structures to optimize its use as a degradable/regenerative scaffold. In addition, individualized constructs can be bioprinted based on information from magnetic resonance imaging and computed tomography. Currently, bioprinting techniques include inkjet, laser-assisted, extrusion, and UV-based bioprinting. These techniques have been described and reviewed in detail elsewhere [1] and is beyond the scope of this review.

This review discusses recent progress in the development of tissue- and organ-derived bioinks for 3D bioprinting. Many researchers believe 3D bioprinting decellularized ECM is a promising strategy for a regenerative scaffold. From a design perspective a 3D bioprinted dECM scaffold has the potential to meet all clinical performance requirements, including some that other bioinks cannot. Several issues that affect the final dECM such as the method of decellularization, choice of tissue or organ used for decellularization, and also the animal from which the tissue or organ is obtained are discussed. Other notable discussions include synthetic materials that can be combined with dECM to make the bioink mechanically stronger. Lastly, the challenges and limitations associated with 3D bioprinting dECM bioinks are discussed.

## 2. Literature Search Methodology

**Figure 2:**
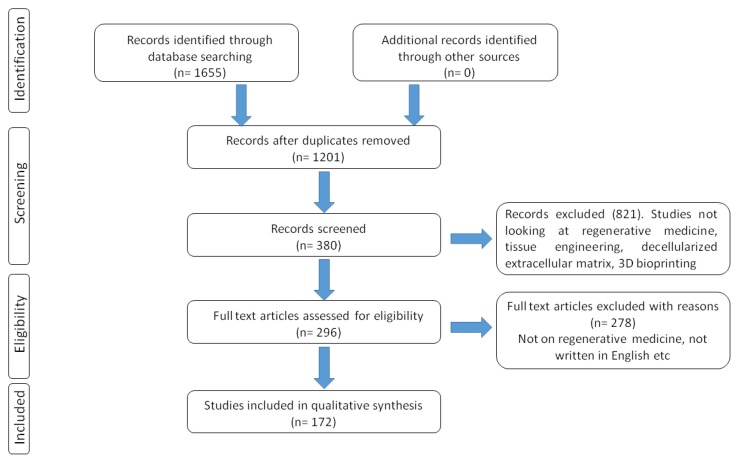
Selection of studies included in the qualitative synthesis of the review manuscript.

A literature search from the PubMed and MEDLINE was performed until July 2019 for relevant articles using the keywords including regenerative medicine, tissue engineering, decellularized extracellular matrix, 3D bioprinting, bioink, cellular reprogramming, scaffolds, biofabrication, personalized medicine, and transplantation. English is the main language in these databases and only full articles were included (Figure 2). A total of 1655 articles were consulted and based on our exclusion criteria, 172 articles were used to gather the relevant information for this review. This literature search methodology allowed us to review information on decellularized extracellular matrix as biomimetic bioinks for 3D printing. Many authors have done great research on regenerative medicine and tissue engineering and we apologize for those not cited in this review.

## 3. Functions of the Extracellular Matrix

**Figure 3:**
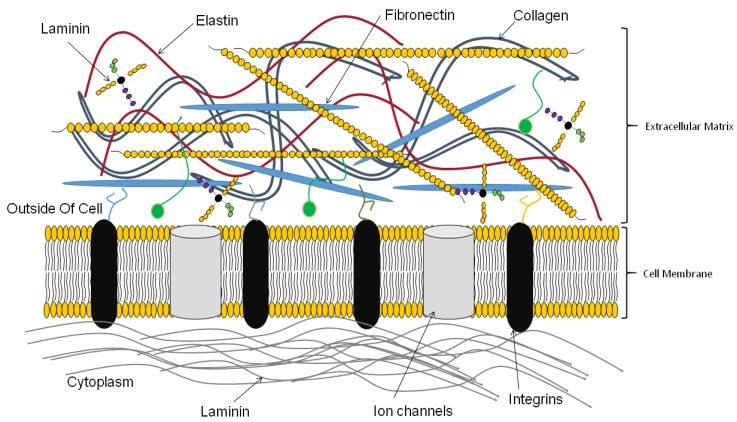
The extracellular matrix.

Tissues and organs contain cells and ECM components such as collagen, fibronectin, laminin, glycosaminoglycans [13,14,15]. The composition of each tissue and organ is specific with cells and ECM mutually interacting with each other. While cells produce the ECM, the ECM in reverse interacts, and influences the behavior of cells [16,17,18]. Cells interact with ECM through cell receptors such as integrins. Cell–ECM interactions activate several signaling pathways important for cellular functions [13,14,15]. Proteoglycans, with attached glycosaminoglycans, are large molecules, occupying more space than ECM fibers. The presence of charged groups especially on GAGs allows the movement of molecules and metabolites in tissues and organs [16,19]. The ECM traps several growth factors, enzymes, and other molecules, thereby influencing and impacting on cellular function and fate [13,14,20,21]. ECM components such as collagen and fibronectin provide the necessary physical strength needed by cells to maintain form and also for migration (Figure 3). Together with other fibrous ECM proteins such as elastin and laminin, they provide the elasticity to the ECM. The specific ECM of each tissue and organ allows cells to perform specific functions. Thus, it is important for scientists to try to recapitulate the native tissue and organ ECM during 3D bioprinting of tissues and organs.

The low regenerative capacity of the human body has seen many attempts being made to improve the body’s regenerative capacity through the use of biomaterials and cellular factors [1,22]. Before complex systems such as cell- or tissue-derived ECMs, extracellular matrix proteins were used as surface coatings. Some of the early products include Biobrane and Integra, used in wound healing and as artificial skin product, respectively. Another skin product, AlloDerm, was obtained from cadavers. Type I collagen mixed with fibroblasts was used to promote healing as Graftskin. Currently, several biomaterials are now known to induce both stem cell differentiation and the release of biochemical factors needed for regeneration and healing processes [23,24,25,26]. Mixed with growth factors, extracellular matrices have been shown to induce the body to create or form tissues and thus can correct defects [27,28,29]. The choice of ECM alone or with cells depends on the regenerative capacity of the tissues being studied. Tissues with low regenerative capacity such as cartilage will require the presence of stem cells whilst other tissues may only require the extracellular matrix [30,31,32].

## 4. Three Dimensional (3D) Bioprinting

**Figure 4:**
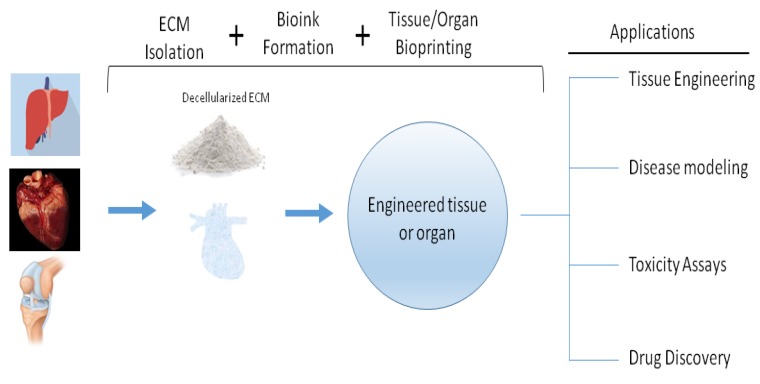
Decellularized ECM can be obtained from tissues or organs. Bioprinting produce engineered tissues or organs that can be used in several applications such as tissue engineering, disease modeling, and drug discovery.

The use of computers to dispense biomaterials, biochemical factors, and cells in a layer by layer fashion in a bid to create engineered tissues and organs is called 3D bioprinting [1,33]. Printed tissues/organs/constructs/patches have several uses in biology and medicine ranging from drug delivery and screening, modeling of diseases and regenerative medicine, and tissue engineering (Figure 4) [34,35,36,37]. Currently, three methods are used during bioprinting and these are light-based (laser- and UV-based), inkjet, and extrusion bioprinting [1,33]. Different estimates have been put forward regarding the global 3D bioprinting market with most estimates of its worthy hovering above US $1 billion [38,39,40]. Novel printing technologies and new products are likely to spur more growth in the field.

**Figure 5:**
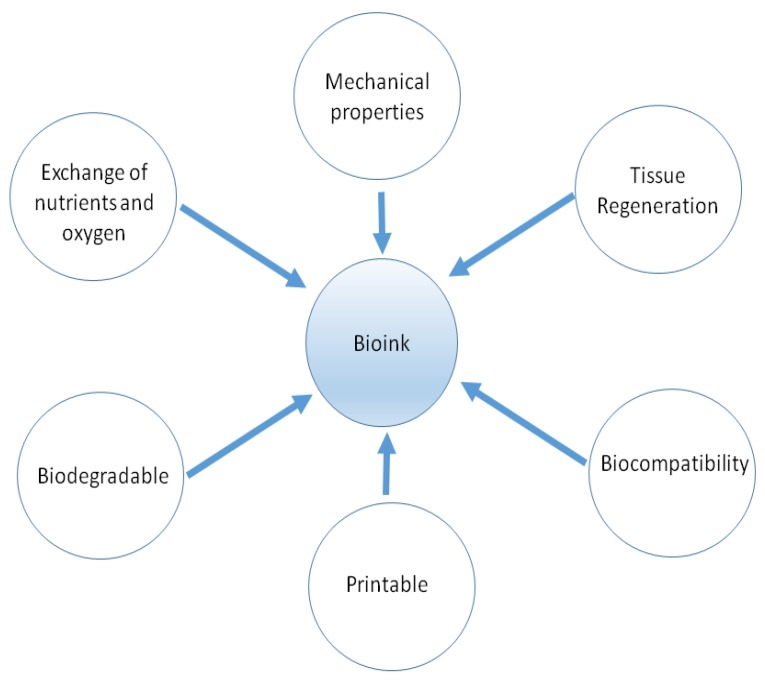
Properties of bioinks to consider for three-dimensional (3D) bioprinting.

3D bioprinting requires bioinks, most of the time made up of soft materials that can be molded and printed with cells [1,16]. Components of the most used bioinks include ECM proteins, cells, and factors such as growth factors. Components of the bioink are mixed in different proportions depending on the end product and coming up with the right mixtures has proved to be a major challenge to bioengineers [41]. This is partly due to the need for the bioink to provide both physical support for cells and also biochemical signals needed by cells for proliferation and differentiation whilst being mechanically stable to be printed [1,33]. Other major challenges regarding bioinks pertain to the need to have a good level of biocompatibility and be able to be printed at a high resolution (Figure 5) [1]. The final bioink mixture must not be poisonous to cells and avoid eliciting an immune response. Much of the bioink will be replaced through tissue ingrowth as the scaffold degrades whilst part of the scaffold can integrate with the host tissue. The properties of the bioink must also allow cells to adhere to it whilst allowing migration to take place [1,16,33]. Once printed, the bioink must maintain the printed structure. Currently, several materials are used as bioinks to print different tissues [35,42]. Naturally-derived hydrogels including ECM proteins such as collagen, hyaluronic acid, silk, and gelatin have been used extensively to print different tissues [16,42]. Hydrogels can support cell growth, are tunable, and can have biodegradable properties. However, due to their low viscosity, gels do not recapitulate some tissues that require specific mechanical properties such as load-bearing properties [14,43]. Tissues requiring high mechanical strength can now be fabricated using synthetic scaffolds including polyethylene glycol (PEG) and polyurethane (PU) [14,43,44,45].

The ECM present in tissues and organs is much complex than the use of individual ECM proteins as done during most bioprinting [1,42,46]. In addition to providing the physical microenvironment within which cells can reside, the native ECM participates in cell–ECM interactions that may be important for proper tissue and organ function [42,47]. Each tissue or organ has a specific ECM with varying composition that supports cellular growth and function. Thus, the use of individual or even mixtures of ECM proteins can hardly recapitulate the native ECM’s properties. The search for the most promising bioink that can induce tissue that approximates the natural ECM to restore function by being a degradable/regenerative scaffold is still ongoing with new and improved bioinks becoming available. A very good promising solution is the use of dECM. dECM is an appealing solution due to the preservation of most if not all functional and structural components of the native ECM [3,23]. dECM can easily form soft gels, a requirement for the bioprinting process. dECMs have also been used as biological sheets and for coating [3,23]. The presence of both physical and biochemical cues allows the dECM to induce restoration of normal tissue or organ homeostasis and cellular processes. What the dECM lacks as a bioink is the mechanical strength needed for the fabrication of load-bearing tissues. Thus, the development of new and improved bioinks is an ongoing research endeavor. Many natural ECM biomaterials and their mixtures are being investigated as bioinks.

As discussed by Dzobo et al., the choice of bioprinting technique employed largely depends on the properties of the biomaterial [1]. For example, inkjet bioprinting requires that the biomaterial to be printed maintain a certain viscosity as the nozzles can be clogged. To achieve this, the number of cells included is usually lowered. This has the effect of producing a construct or product with fewer cells than maybe required. Inkjet bioprinting droplets can be approximately 50 µm in diameter [48]. Although micro-extrusion can bioprint bioinks with high number of cells compared to inkjet printing, final cellular viability tends to be lower for micro-extrusion bioprinting, limiting its use when high cell density is required. Laser-assisted bioprinting can print bioinks with cell densities close to physiological tissues or organs. However, laser-assisted bioprinting generates metallic residues within the final construct or product. UV-light-based bioprinting affect the viability of cells, with final cellular numbers lower than physiological tissues and organs. In order for dECMs to be printed properly, there might be a need to modify the dECMs before or during printing to avoid the above-mentioned problems.

## 5. Preparation of dECM

dECMs maintain the ECM composition of the native tissue or organ, thereby providing tissue- or organ-specific microenvironments for cellular growth and function. Several scaffolds made of dECM have been developed and have been shown to contain many biological molecules including growth factors, cytokines, and microRNAs [16,17,49,50,51]. Certain requirements must be fulfilled by the dECM for successful transplantation. The amount of residual DNA left behind after the decellularization procedure must be below 50 ng dsDNA/mg weight. The decellularization procedure must be gentle to avoid damaging the ECM and therefore affect its composition.

### Decellularization Methods

The ultimate goal of a decellularization step is the removal of cellular components whilst maintaining the native structure and composition of the tissue or an organ. Thus, the choice of decellularization method plays a key role in determining the final properties of the dECM bioink obtained. The decellularization procedure involves cellular lysis followed by separation of cells from the ECM. Several physical, chemical, and biological methods are used during decellularization and the choice of which one to use depends on several factors such as tissue and organ thickness, density, and also lipid content (Table 1) [14,52]. Several acids, bases, detergents, and alcohols are some of the chemicals that have been used before whilst enzymes such as trypsin and nucleases constitute the biological methods available to date [15,52]. Decellularization can also be achieved through the use of other methods such as sonication, heating, applying pressure, and electroporation [53].

Chemicals are frequently used in decellularization procedures. Ammonium hydroxide, SDS, and Triton X-100 are some of the chemicals commonly used during decellularization of both tissues and organs, yet are known to remove or damage ECM components [52]. Acid and base treatments including the ammonium hydroxide and sodium hydroxide can result in removal of growth factors and can affect the strength of the final dECM. One advantage of using acid and base treatments is the sterilization of the final dECM. Triton X-100 is known to decrease the amount of fibronectin and laminin in the final dECM. Triton-X-100 is known to damage the ultrastructure of the ECM in addition to the removal of some glycosaminoglycans (GAGs) [61,65]. Triton X-100 was shown to leave large quantities of cellular material when used for decellularizing the tendon [64,66,67]. Ammonium hydroxide is one common method used for cellular removal, though it is known to induce some damage to ECM collagens [68,69,70,71]. Growth factors are known to be sensitive to the presence of detergents such as SDS [52]. SDS has also been shown to damage ECM proteins such as collagen [61]. An advantage of using SDS is that it can be used for dense tissues and organs. Enzymatic decellularization can be achieved through the use of nucleases (DNase, RNase) and proteases (Trypsin, Dispase). Exposure of the tissue and organ to trypsin for too long can result in damage to the structure and dECM bioinks lacking ECM proteins such as collagen, fibronectin, and elastin [15,52,61]. Nucleases are known to decrease the amount of collagen in the final dECM in addition to the difficulty associated with their removal from the tissue or organ. Physical decellularization can be achieved through the use of freezing, osmosis, agitation, and direct pressure. Though there is less disruption of the ECM, physical decellularization can result in incomplete removal of cellular debris, which can cause immune reactions if dECM is used for transplantation.

Large organs are usually decellularized through a perfusion pathway. The decellularization agent is added through the vasculature [72]. dECM bioinks only require the maintenance of ECM composition and therefore tissue and organs can be cut, sliced or ground into small pieces [73,74]. The pieces are then exposed to the decellularization agent with shaking for different periods of time ranging from hours to days and sometimes weeks [14,42,52,61,75]. In addition, cutting or slicing the tissue or organ into small pieces allows the process of decellularization to be done in a short time due to increased surface area [61]. Dissolution, however, is one major disadvantage of using small pieces of tissue or organ.

Several chemicals, alcohols, and acids have been used during the sterilization of the dECM bioinks. Ethanol (4% *v*/*v*) and peracetic acid (0.1% (*w*/*v*)) are some of the commonly used sterilization agents. Gamma irradiation is also used for sterilization of dECM. Due to the viscous nature of dECM bioinks, filters do not work very well, though they have been used [76]. Alternative ways to sterilize the bioink include the use of ethylene oxide gas and carbon dioxide [77,78,79]. One major problem associated with the sterilization step is the potential disruption of the final dECM. High dosages of gamma irradiation can cause changes to the strength of the dECM.

## 6. Decellularized ECM as Bioink

In vitro recreation of both tissues and organs require the use of specific biomaterials [15,52]. Thanks to recent advances in decellularization techniques, decellularized ECM can now be used in the in vitro reconstruction of tissues and organs. The resulting decellularized ECM can be used as is, as a patch, as a gel or be reduced to powder form before utilization [1,80]. Surgical mesh biomaterials are mostly made from decellularized tissues of different animals tissues [52]. Sources of tissues include the skin, small intestines, and urinary bladder [52]. Different dECMs will have specific effects on different cells, with recent advances including the addition of synthetic polymers to dECMs, fine-tuning them to certain specifications as required [81]. Currently, several tissue models have been bioprinted using tissue- or organ-derived dECM bioinks. dECM bioinks derived tissues and organs including liver, heart, adipose tissue, cartilage, and skin [47,50,82,83]. The presence of biomolecules such as growth factors and other factors allow the proliferation and also differentiation of cells if stem cells are used [14,50,83,84]. Growth factors are especially important in cell to cell communication, influencing proliferation, migration, adhesion, and cellular differentiation. The fabrication of these tissue constructs has made significant contributions to science in several areas such as disease modeling, drug screening, and regenerative medicine. One major issue still to be resolved is that of a good blood supply. In order for the new tissue or organ to survive or be incorporated into the surrounding tissue, there is a need for adequate vascularization.

One major source of dECM bioinks is the pig [74,76,84]. The use of porcine tissues and organs is superior to other animals’ tissues and organs in several ways. Porcine organs are easily obtained and available in large quantities than those from other animals [14,85]. Due to their higher breeding ability and the high number of offspring, pigs have been the number one choice when it comes to providing tissues and organs for dECM bioinks [86,87]. One human tissue normally used for the production of dECM bioinks is adipose tissue. Patients who normally undergo liposuction produce a lot of medical waste in the form of adipose tissue. Decellularized human adipose tissue was used to design and print dome-shaped structures with engineered porosity [84]. The human dECM bioink induced high cell viability over a period of time and also induced the expression of several adipogenic proteins with no supplementation of adipogenic factors [84]. Overall, human adipose tissue can be a source of dECM bioinks and also stem cells [1,84,88,89]. Rat and goat tissue and organs such as adipose tissue, heart, and liver are additional sources of dECM bioinks [90,91,92]. It is important to point out that most of the dECM bioinks from animals such as rats and cows are mostly used for research purposes.

Major challenges exist on the use of porcine tissues and organs. The potential of infectious disease transmission exists and can be minimized through having pigs bred in a controlled manner and environment. It is known that several viral genomes are integrated in animal genomes, making the use of animal tissues and organs a risk [93,94]. The risk of infectious disease transmission has rarely if ever been established following the use of xenogeneic ECM sources. Another challenge involves the possible immunological reaction to the presence of the animal dECM. Several animal antigens are recognized and can result in the remodeling of the dECM [95]. Latest gene editing and cloning techniques can prevent the immune reaction altogether via knocking out the antigen gene in the animals. Such antigen gene-knockout animals have already been produced, resulting in the formulation of dECM bioinks that causes no immune reaction [95,96]. Several studies have produced both animal and human dECM bioinks and these are described below.

Jang et al. used the porcine left ventricle tissue to produce dECM bioinks, which was able to recapitulate cardiac tissue and induced high cell viability and proliferation of human cardiac progenitor cells [76]. In addition, the dECM bioink was able to induce human cardiac progenitor cells to express several transcription factors important in cardio-myogenic differentiation [76]. The authors employed sodium dodecyl sulphate (SDS) and Triton X-100 during the decellularization process. To increase the mechanical properties of the dECM bioink, the authors utilized vitamin B2 and UVA light to enhance crosslinking of the dECM bioink. Sterilization of the porcine tissue was done using 0.1% (w/v) peracetic acid for several hours [76]. In another study, Pati et al. also used the porcine left ventricle tissue to print a cell-laden construct that provided a microenvironment allowing the growth of three-dimensional structured tissue [97]. The authors successfully decellularized not only the porcine heart tissue but also adipose and cartilage tissues using SDS and Triton X-100 [97]. The heart dECM bioink obtained contained no synthetic supporting polymers. Overall the authors were able to show that dECM bioinks obtained from different tissues including adipose and heart tissues are capable of directing cellular engraftment, proliferation, and survival for long periods of time [97]. Recellularization of the decellularized heart tissue can be achieved through the use of mesenchymal stem cells from cord blood, bone marrow, and cardiac progenitor cells [98,99]. Cartilage, a connective tissue necessary for body parts movement, can easily be damaged. Cartilage exists as a rubbery tissue which cushions the bones during movement. Cartilage defect is a common disorder experienced by many people, resulting in intense joint pain and stiffness. One possible way to correct or repair the cartilage is through the use of biomaterials and/or stem cells. Pati et al. derived cartilage dECM bioinks from porcine cartilage and showed that the cartilage dECM bioink induced enhanced chondrogenic differentiation of human stromal cells than collagen bioinks [97]. At present, several techniques are being used to treat cartilage defects and these include autologous chondrocyte implantation and matrix-induced autologous chondrocyte implantation [100,101].

Liver disease affects millions of people worldwide with many going without cure due to shortages of donors. Depending on the source of information, about 80–100 millions of Americans suffer from the non-alcoholic fatty liver disease [102]. In the UK alone liver disease is known to affect more than two million people [103]. Alternative treatment options are being sought including regenerative medicine and tissue engineering strategies. Skardal et al. derived dECM bioinks from porcine liver and showed that both biochemical and physical factors are important in cellular behavior and differentiation in vivo and should be considered together during in vitro fabrication of tissue constructs [74]. The authors incorporated tissue-specific biochemical factors in the bioink and this was important in directing tissue-specific cellular growth and differentiation [74]. For decellularization, the authors used both Triton X-100 and ammonium hydroxide (NH_4_OH). To obtain their desired dECM bioink stiffness, the authors utilized polyethylene glycol-based crosslinking, allowing the printing of bioink at specific speeds. Another study by Lee et al. also derived dECM bioinks from porcine liver [73]. Decellularization was achieved via the use of Triton X-100 and SDS. The above study showed that stem cell differentiation and liver cell functions were much enhanced using the liver dECM bioink than other commercially available bioinks such as collagen bioinks [73].

Many people, especially sports people, will suffer muscle injuries at one point in their lifetime. Several treatment strategies exist with the engineered muscle tissue being one of novel strategies considered for muscle injury treatment. Choi et al. were able to print dECM bioinks derived from porcine skeletal muscle, which provided a myogenic environment to myoblasts, allowing high cellular viability, and contractility [104]. In addition, the dECM bioink was able to enhance the formation and maturation of myotubes than a collagen bioink and responded well to electrical stimulation [104]. Ahn et al. derived dECM bioinks from porcine skin tissues, with their method showing the retention of collagen from the native tissue [75]. The authors showed that their method of bioprinting, involving a heating system, did not adversely affect cell viability whilst ensuring gelation of dECM bioinks [75]. About a tenth of the human body mass comes from the skin, its largest organ. The skin has an incredible regeneration capacity. Whilst minor injuries are easily repaired, chronic wounds are very costly and cause a lot of pain to the patient. The loss of fibroblasts proliferation abilities, low levels of growth factors, and the loss of the ECM are some of the major causes of chronic wounds [105]. Currently no skin substitute meets all performance requirements and research into developing one is ongoing with focus on the use of human derived ECM. Several issues such as vascularization and scaling must be addressed first. Available human skin regeneration products are based on the decellularization of animal tissues. Despite proving effective at increasing fibroblast and keratinocyte proliferation and migration, these animal-derived ECMs are not ideal for human use and do not allow scar-free healing [106,107].

Lastly, of the few recorded studies involving human tissue, Pati et al. derived bioinks from the human adipose tissue and showed that it is capable of providing biochemical and physical cues for cells to proliferate, differentiate, and survive for long periods of time [97]. The human adipose tissue was decellularized through the use of SDS and produced bioink, which was able to induce adipogenic differentiation of human mesenchymal stromal cells more than collagen bioinks [97]. In another study, Pati et al. injected bioinks from the human adipose tissue into nude mice [84]. The authors showed that the bioink allowed increased cell viability and expression of adipogenic genes with no addition of adipogenic factors [84].

To avoid disease transfer from animals, human tissue would be the best to use for deriving dECM bioinks for clinical applications. Several studies have, however, shown that animal tissue can perform better than human tissue in terms of stability and induction of stem cell differentiation [108,109].

## 7. dECM Bioinks Modifications

The native ECM has both structure and order, with several proteins, proteoglycans, and glycosaminoglycans intricately placed in a certain fashion [80]. The ECM is tissue-specific and plays key roles in cellular proliferation, anchorage, migration, and signaling [110]. The mixing of different ECM proteins to produce arrays, in a bid to recapitulate the in vivo ECM, has been and can be used to study tissue-specific cellular processes. Using ECM arrays from porcine tissues, Beachley et al. evaluated how different cells including stem cells and immune cells would respond to tissue-specific ECM arrays [110]. Their results show that many ECM proteins can be associated with specific cellular functions. The same study revealed that growth factors differ between different tissues and certain ECM proteins are more abundant in certain tissues. Similar studies have been performed using cell-derived ECMs and shown that ECM proteins are essential for cancer cell migration and survival [111]. Thus, it is important to choose the right organ during dECM bioink manufacturing.

In addition to differences in the ECM composition of tissues and organs, individual animals will have distinct compositions in the same tissue or organ. Several modifications can be done on dECM bioinks before and after bioprinting. Since dECM bioinks are soft it is likely that the 3D orientation of active sites of native ECM is preserved, thus the addition of several chemical and biological crosslinking agents may help to improve the mechanical strength and bioactivity of the final scaffold. For example, vitamin B2 can crosslink collagen in the presence of ultraviolet A light [50,112]. Polyethylene glycol diacrylate (PEGDA) has acrylate groups able to crosslink with each other, thereby trapping the dECM bioink, making the dECM bioink mechanically stronger in the process [74]. Cells must be protected if they are printed together with the dECM bioink. Parameters such as viscosity will affect printing speed and the final resolution of the dECM bioink. Mixing of dECM bioinks with nanofibrillated cellulose has been suggested as a way to increase printing resolution [73]. Parameters such as gelation mechanism and printer nozzle size and diameter will affect the printing speed [73,76]. The printed dECM bioink must be able to maintain its structure after printing. It is important that the viscoelastic properties of the printed dECM bioink recapitulate that of the native tissue or organ [14,23,113,114]. For some applications, the composite of the bioprinted ECM and the ingrown tissue should approximate the mechanical properties of the native ECM. The properties necessary for a scaffold are likely to be different than that of the native tissue as the scaffold has to stimulate cellular proliferation and migration. dECMs can also be modified via methacrylation. The chemical-modified dECM can then be mixed with other proteins such as hyaluronic acid and gelatin.

## 8. Cell-derived ECMs Versus Tissue/Organ-derived ECMs

dECMs obtained from tissues and organs are more complex and contain far more factors such as growth factors than cell derived ECMs [14]. The source of the dECM used during bioprinting will influence the quality and composition of the printed scaffold. Tissue or organ dECMs also recapitulate the native tissue or organ better as they have both architecture and mechanical properties. One major drawback of tissue and organ-derived dECMs is availability or lack thereof. Tissue or organ dECMs are also likely to be different as the tissue or animal of origin age and if the gender is different. Stem cells occupy specific regions of tissues and tissue and organ ECMs may not represent stem cell niches [115,116]. Tissue- and organ-derived dECMs also present problems when it comes to large scale in vitro analysis, something that is possible with cell-derived dECMs. Thus, in some instances, cell derived ECMs may be better than tissue or organ derived ECMs [1,89,107]. When cells are cultured, they synthesize, secrete, and assemble ECM components around them. Once enough ECM is deposited the cells can be removed through decellularization. Thus, cell derived ECMs are easily obtained through culture of cells and can be scaled up through the culture of cells in bioreactors, for example. Most importantly, cells in culture can be manipulated through gene knockdown and overexpression to control the amount of ECM proteins synthesized [14,107,111]. Mesenchymal stem cells, cells that can be obtained easily, are commonly used to make cell derived ECMs. Such mesenchymal stem cell-derived ECMs are able to maintain stem cells in their undifferentiated state in vitro [17]. Several studies have shown that fibroblasts can produce ECMs rich in collagens type I, II, III, and fibronectin [89,111,116,117]. With so many advantages over tissue or organ derived ECMs, cell derived ECMs are an appealing source of dECM bioinks. One major disadvantage of cell-derived dECMs is that their composition and mechanical strengths might be slightly different from the native ECM [118]. Both cell-derived and tissue/organ-derived ECMs have been used is several studies and have shown the capability to direct cellular behavior (Table 2).

A fibroblast-derived extracellular matrix (fd-ECM) was able to induce chondrogenic differentiation of adipose-derived mesenchymal stromal cells (ad-MSCs) in vitro [89]. Although the fd-ECM reduced ad-MSC proliferation, it induced chondrogenic differentiation of the ad-MSCs through the β-catenin signaling. In addition, the fd-ECM showed anti-senescence effects on ad-MSCs, making it a promising approach for the induction of chondrogenic differentiation of stem cells [89]. However, another study showed that fd-ECM was able to induce proliferation of MC3T3-E1 cells whilst an osteoblast-derived ECM was able to promote osteogenic differentiation of the same cells [129]. An ECM derived from mechanically stretched cardiac fibroblasts was shown to improve the metabolic activity of ventricular cells [130]. The same study showed that a proteoglycan-attached glycosaminoglycan was responsible for the observed effect [130]. It has been shown that the application of skin products containing human fibroblast-derived growth factors can result in significant upregulation of genes encoding ECM components including collagens and elastin [131]. Human lung fd-ECM was incorporated into a collagen hydrogel and was shown to provide an angiogenic microenvironment for HUVECs [132]. In addition, the authors observed a synergistic effect of fd-ECM and angiogenic growth factors in the 3D construct [132].

## 9. Other Bioinks Utilized in 3D Bioprinting

To present a balanced review of different bioinks used in 3D bioprinting, we discuss the use of several polymers for bioprinting. We acknowledge the development of new and improved ECM-like bioinks with properties usually absent in dECM-free bioinks. For example, the combining of different bioinks during bioprinting, including combining dECM and polymers, can result in bioinks that can form fibrillar networks as well as bioinks that can sequester/release growth factors [133,134]. The net effect of combining bioinks will be to improve the biochemical and biological properties of the final bioink, with the goal of better tissue biomimicry compared to individual bioinks. These new and improved ECM-like bioinks are under intense investigations and are beyond the scope of this current review.

Agarose is able to form gels easily and has been used in various applications in laboratories worldwide. Agarose has very good gelation properties and is biocompatible. One major limitation to the use of agarose as a bioink pertains to its reduced cell growth promoting property [135]. As a result, bioinks made up of mixtures of agarose and other proteins including collagen and fibrinogen have been developed [136]. The agarose-based bioinks were able to form mechanically stable structures in addition to supporting cellular growth. A mixture of agarose, collagen, and sodium alginate was used as a bioink for cartilage tissue engineering, with results showing increased mechanical properties compared to either biomaterial [137]. Agarose together with alginate and carboxymethyl-chitosan was successfully used to produce constructs able to form functional neurons from induced pluripotent stem cells [138,139,140].

Another attractive polymer for use as a bioink is alginate. Sourced from brown algae, alginates are known not to induce an immune response when used during transplantation. Being able to encapsulate several molecules, alginate can therefore be used to deliver biomolecules and other factors [141]. In addition, alginate-based bioinks can form hollow structures, making them useful in making microfluidic chips [142,143,144]. Alginate when used together with cartilage progenitor cells formed tubular structures able to support cartilage progenitor cell growth [145]. One of the major challenges faced by scientists is the lack of vascular vessels in constructs. A sodium alginate bioink containing fibroblast cells was able to form vascular structures via the use of a calcium chloride crosslinker [146]. Several reports show that alginate can successfully be used as a bioink in different applications. Faulkner-Jones et al. used alginate as a bioink to study the differentiation of embryonic stem cells into hepatocytes-like cells [147]. Incorporation of other compounds such as nanosilicate clays is known to enhance printability and biocompatible properties of alginate. A combination of alginate and polymers such as gelatin methcryloyl (GelMA) can be used for vascular tissue engineering [148].

As the main component of the ECM, collagen has attracted the attention of scientists and has been used as a bioink alone or in combination with other ECM proteins [149,150,151]. Several studies have shown that collagen can be modified through the use of vitamin riboflavin as well as temperature, resulting in enhanced tensile and viscoelastic properties [152,153,154,155]. Collagen gelation takes more time limiting its use in 3D printing. The use of a bioink consisting of collagen together with sodium alginate produced a much stronger construct that was effective at preventing the transformation of chondrocytes and increased cell proliferation [137]. This resulting construct can therefore be used in cartilage tissue engineering applications. A bioink made up of a mixture of collagen and gelatin produced 3D constructs with enhanced biological activity, with cells able to cell spread nicely and proliferate more than the use of individual polymers [156]. A combination of collagen and alginate together with adipose stem cells showed enhanced cell viability and growth compared to individual polymers [157]. Gelatin crosslinked with transglutaminase produced a construct able to develop vascular networks and can be used in many applications from cancer studies to tissue engineering.

Found predominantly in cartilage and connective tissues, hyaluronic acid has been used as a bioink in 3D printing to construct structures for the same tissues [158]. Like collagen, hyaluronic acid gelation takes time and therefore combinations of these polymer and others have been sought. Hyaluronic-based hydrogels have been developed through modification with methacrylate and these hydrogels demonstrate increased osteogenesis inductive behavior [159]. A hydrogel made up of hyaluronic acid, polyglycidols together with polycaprolactone enhanced chondrogenesis [160]. Furthermore, combinations of hyaluronic acid and gelatin have been used as bioinks to produce constructs that can promote cell viability and when needed, differentiation of stem cells [161]. Several other bioinks used in 3D printing include cellulose, fibrin, silk, and cellular aggregates. The enzymatic treatment of thrombin results in fibrin hydrogel that has shown relatively good biocompatibility [162]. Fibrin-based bioinks have been used to make constructs for urethra and nerve regeneration. Cellulose is converted to carboxymethyl cellulose before being used as a hydrogel [163,164]. A nanocellulose and alginate bioink was used to make constructs for cartilage tissue engineering [165]. Nanocellulose hydrogels can also be used to make patient-specific cartilage tissue, with the constructs displaying good mechanical strength and cell viability when printed [166]. In addition, bioinks can be made from silk, cellular aggregates, and synthetic biomaterials.

## 10. Perspectives

### 10.1. dECM Bioink 3D Bioprinting

Due to their viscous nature, dECM bioinks have been printed mainly using the extrusion-based bioprinting method. The extrusion-based bioprinting method is easily adapted for different types of dECM bioinks, very cheap to maintain and can print bioinks with high number of cells and can print porous scaffolds [33,167]. Several adaptations of the extrusion-based bioprinting method have been utilized to date. A multi-head tissue/organ building system (MtoBS), with six nozzles, was used to print several constructs including the heart and cartilage [84,97]. Two of the nozzles were specifically for extruding thermoplastic filaments whilst the other four were for extruding bioinks or hydrogels [84,97]. A major disadvantage of the multi-head printing system is the need to print one material at a time but there is no limit to the number of biomaterials that can be printed. This slows down the printing process as well as affects the integration of the different materials printed. Cardiac tissue constructs and a stem cell patch were also bioprinted by Jang et al. using the MtoBS [104]. Other adaptations of the extrusion-based bioprinting method include the integrated composite tissue/organ building system (ICBS), used to bioprint skeletal muscle dECM [104]. The inclusion of two heating modes is yet another adaptation of extrusion-based bioprinting that allows for precise stacking of dECM bioinks [14]. Other printing methods such as ink-jet and laser-assisted bioprinting can also be used to bioprint dECM bioinks [33,168]. Microfluidics can be used to print different bioinks. A microfluidics system can mix different bioinks and cells into droplets. Microfluidic bioprinting is one of the fastest printing methods available to date and has been shown to print seven different types of materials [169]. Currently most multi-bioprinting strategies suffer from reduced printing resolution, lack of or reduced integration of biomaterials, as well as, reduced mechanical strength or the final construct or product. In addition, vascularization is still an unresolved issue with all 3D bioprinting technologies as without adequate blood supply there won’t be integration into the surrounding tissue or healing at the interfaces.

### 10.2. Manufacturing and Regulatory Process Considerations

Many factors need to be taken into consideration if bioprinting of dECM bioinks is to be scaled up to the industry level. Several companies are currently involved in producing 3D bioprinters and commercializing the printing of large tissues. These include CellInk and Organovo [170,171,172,173,174,175,176]. Though faced with many challenges including scaling up of the processes, regenerative medicine technologies are expanding and are likely to impact on patients’ treatment in future. In addition to the choice of tissue/organ, decellularization method, sterilization, factors such as the cost, yield of dECM bioink, quality and batch size must also be taken into account. For example, besides waiting to get the right tissue/organ from an animal, decellularization can take weeks. Optimization must be done to come up with the best method for most steps involved in the manufacture of the dECM bioinks. The state of the tissue or organ is very important as any delay in decellularization can result in changes in the ECM composition of the tissue or organ. It is recommended that fresh material be used all times. Before gelation of dECM bioinks, mass spectrometric and rheological analyses can be performed to identify the specific protein composition of the bioink and to measure the amount of time and bioink speed needed for gelation, respectively. Commonly used methods to ionize proteins include matrix-assisted laser desorption/ionization (MALDI) and electrospray ionization [111,177,178]. Proteomic analyses performed on several ECMs (from tissues/organs or cell-derived ECMs) show that most animal ECMs contain collagens, proteoglycans, glycoproteins, ECM regulators, and ECM-affiliated proteins in various amounts [14,85,111]. Mass spectrometric analysis of ECMs can also be combined with 2D gel electrophoresis in order to screen the mixture of proteins and other factors present in tissues and organs. dECM bioinks or gels needing mechanical strength can be mixed with a crosslinking agent during the bioprinting process [76].

Parameters such as viscoelaticity and elastic modulus of dECM bioink determines stem cell differentiation, thus it is important that dECM bioinks’ elastic modulus is determined or controlled through the use of crosslinking agents [76]. The efficiency of the decellularization step must be determined through testing for residual cellular material. One such test is checking for cellular DNA. Small amounts of DNA such as 50 ng DNA are usually considered as decellularized properly [52,97,179,180,181,182]. The amount of collagen and other ECM components can also be determined through assays such as the hydroxyproline assay [179,180,181,182]. The bioprinting process can adversely affect cells and therefore cell viability assays must be performed. Several assays are available to achieve this including the use of ethidium homodimer staining [104]. To verify stem cell differentiation, RT PCR can be performed to evaluate gene expression [84].

Cells such as fibroblasts and keratinocytes have been utilized together with collagen for the treatment of venous and foot ulcers [183,184]. Cell-derived ECM has also been shown to induce wound healing and in vitro cartilage formation [1,89,185,186,187]. The inclusion of a cellular component would allow the release of growth factors and cytokines to help with the regeneration or healing process [89,183]. Millions of people are affected by cartilage problems or disorders at some point in their lifetime, resulting in careers being cut short or lives dramatically changed as movement is affected [188,189,190]. Cartilage defects are caused by trauma, diseases, and degenerations over time [191,192]. Currently, cartilage damage or defect is treated through many strategies including drilling into the bone to release stem cells that can help heal the damage. Some of these processes are too invasive. Regenerative medicine using 3D printing of extracellular matrix with stem cells is an increasing viable option to repair and replace diseased and damaged tissues such as cartilage. Collagen matrix combined with chondrocytes, marketed as matrix-associated autologous chondrocyte implantation (MACI), was approved by the FDA for knee cartilage defects [2,6,193]. The use of decellularized ECM for cartilage treatment is hindered by the need for compact cartilage tissue. However, scientists have succeeded in using decellularized cartilage tissue, albeit with some modifications of steps such as decellularization [194]. By reducing the exposure to decellularization agents, the structure and composition of the ECM is maintained [195,196]. Recellularization of the dECM is achieved through the use of mesenchymal stem cells and in some cases primary cells such as chondrocytes [197,198].

One of the major challenges faced by the field of regenerative medicine and tissue engineering is that of tissue or graft integration, or the lack thereof. To improve integrations and also be able to create organs, scientists have sort to create vascularized tissues or grafts. dECMs bioinks have tremendous potential in several tissue engineering applications due to their preservation of specific binding domains and potential maintenance of functional vasculature. 3D bioprinting utilizing more than one cell type provides an opportunity to print vascularized tissues due to proper and precise placement of cells and matrix [199]. Advanced bioreactors are allowing the differentiation of large quantities of cells for use as sources of controlled differentiated cells, growth factors, and cytokines [200,201]. Enabling technologies such as gene editing, via the use of CRISPR-Cas9 and siRNA technologies, together with bioreactors can produce large quantities of specific and modified cell types for specific purposes such as production of growth factors. Vascularized tissues are being produced, providing an opportunity to create complex tissues and organs [202,203]. New and improved ECMs or scaffolds such as biodegradable ECMs and polymers are available for many applications including cartilage and spinal cord repair [204,205,206]. Cells can deposit ECMs on scaffolds and later be removed through a decellularized process, leaving a graft that can be used for several applications [207,208,209].

Several natural ECMs have been approved by the Food and Drug Administration (FDA) for use as therapies for diverse conditions as wound healing and liver regeneration [210,211,212,213,214]. Efforts to get regenerative medicine technologies to patients fast are hampered by regulatory requirements as stipulated by the FDA. The flourishing of illegal stem cell clinics has not helped as well, in a way tainting the whole field and raising suspicions on what is effective and what is not. In addition, stem cell tourism in countries where regulatory oversight is relaxed or absent continues unabated. Several discussions have taken place on the topic of stem cell and ECM use in regenerative medicine applications, with hope that in future therapies and products shown to be safe can be approved without delay [6,215]. One area that might require expedited approval is the use of autologous stem cells and ECMs [2].

The use of extracellular matrices has expanded to include fields such as cancer and disease modeling [216,217,218,219]. Differentiated stem cells, encapsulated in ECM, have been used in the production of insulin, opening up a way for the treatment of diabetes [220,221]. Recently a mobile printing system was developed to treat chronic wounds caused by diabetes and burns, right at the clinic bedside [222]. Chronic wounds require multiple treatments over time and can be fatal and very costly. Early treatment is vital for the patient’s survival. The mobile bioprinting technology can be used for the treatment of extensive wounds through deposition of fibroblasts and keratinocytes directly onto the wound [222]. Combining bioprinting with an advanced imaging system allowed the printer to deposit cells and hydrogels at the precise location on the wound, replicating the structure of the skin. The authors used porcine and mouse preclinical animal models and showed that the rapid management of a wound using this mobile system accelerate the formation of skin [222]. Such novel strategies will continue to be developed and the future looks bright for the regenerative medicine field. Many studies utilizing animal studies and hydrogels get exciting results. However, extension to humans in clinical trials can result in failure. This is why the use of human decellularized ECM would give better clinical results. In addition, there is a need for proper sequential analysis of the wound healing process to avoid undesirable growth of tumors or to avoid scarring. Methods such as microdissection can be used to selectively and routinely investigate and follow the wound healing process at single cell level. This is important in order to obtain relevant and specific data about the wound healing process. The critical thing is to get more regenerative healing and less scar healing. Combinations of ECM and cells have been used to model cancers as cancer-on-chip systems in order to try to recapitulate cancer cell response to drugs for example [223,224]. The cancer-on-chip system is very appealing as it will limit the use of animals as models to study cancer. The use of growth factors in the field of regenerative medicine and tissue engineering is one that requires further investigations. Used mostly to induce and promote cell growth and differentiation, growth factors can have severe side effects and case toxicity [225,226]. Several alternatives have been suggested including the use of medicinal remedies and the optimization of the amounts of growth factors to be used [2,227]. The use of latest technologies such as artificial intelligence in regenerative medicine and tissue engineering is likely to improve the qualities and properties of the bioinks and biomaterials being produced, with the ultimate goal of improving patients’ lives [222].

## 11. Conclusions

The goal of tissue engineering/regenerative medicine is to restore function to diseased or damaged tissues and organs. Currently we normally use grafts, internal or external devices, or pharmaceuticals to restore as much function as possible. In most cases, man-made graft substitutes do not perform as well as the current treatments. A successful strategy has been to make degradable/regenerative scaffolds. Making the scaffold out of the ECM from the area to be replaced has shown improvements over other biomaterials, because it has similar bioactivity to the native tissue. Using the ECM as a bioink, from the dECM of similar tissue, to 3D print the scaffold allows the ability to tailor to the individual case. 3D bioprinting also allows the ability to incorporate cells and biochemical to further enhance the scaffolding ability.

As with all materials made from biologics, there are batch to batch variations in dECMs and therefore bioinks made from them. Enabling technologies such as 3D bioprinting, design technologies, and even artificial intelligence are likely to aid in the development of tissue constructs that can be used on humans. The ECM is very resistant to degradation allowing even cadavers to be used as sources of tissue for tissue engineering purposes. The availability of different types of stem cells such as induced pluripotent stem cells and mesenchymal stem cells is likely to spur more research into the recellularization of dECMs.

## Figures and Tables

**Table 1:** Various decellularizing strategies used to produce dECMs.

Method	Strategy	Material Utilized	Mechanism	References
**Biological**				
	Enzymatic	Nucleases	Breaking up ribonucleotide and deoxyribonucleotide chains	[53,54,55,56]
		Trypsin	Cleavage of peptide bonds between Arginine and Lysine	[54,56,57]
**Chemical Agents**				
	Acid	Acetic acid	Denaturation of proteins and solubilisation of cytoplasmic components	[53,54,56]
	Bases	Sodium Hydroxide, Calcium Chloride, Magnesium Sulphate	Nucleic acids disruption and protein denaturation	[1,53,54,56,58,59]
	Chelating Agents	EDTA	Disruption of cell adhesions	[54,56]
	Hypotonic Detergents	Tris-HCL	Osmotic shock and Protein–DNA disruptions	[53,60,61]
	Ionic Detergents	SDS	Solubilisation of the cytoplasm and nucleus	[61,62]
	Non-ionic detergents	Triton-X-100	Disrupt protein–lipid and lipid–lipid interactions	[1,53]
**Physical**				
	Freeze–Thaw	Liquid Nitrogen	Ice crystals breaks up cell membrane	[63,64]
	Agitation		Cellular Disruption	[61]

**Table 2:** Sources of cell-derived and tissue/organ-derived extracellular matrices and their effects on cells.

**Cell-Derived Extracellular Matrix**		
**Source**	**Effect on Cells**	**References**
Bone Marrow Mesenchymal Stem Cell–Extracellular matrix	Promotion of cellular proliferation and stemness	[17,119]
Fibroblast-derived-Extracellular matrix	Chondrogenic differentiation of cells	[89,120,121]
Placenta Mesenchymal Stem Cell–Extracellular matrix	Osteogenic Differentiation of cells	[122,123]
**Tissue/Organ-Derived Extracellular Matrix**		
**Source**	**Effect on Cells**	**References**
Bovine myocardial Extracellular Matrix	Myogenic Differentiation	[124]
Cartilage Extracellular matrix	Chondrogenic differentiation	[125,126]
Bladder Extracellular matrix	Promotion of cellular proliferation and stemness	[127,128]

## Abbreviations

ADSC	adipose derived stem cell
ECM	extracellular matrix
ESC	embryonic stem cell
FDA	Food and Drug Administration
FGF	fibroblast growth factor
HA	hyaluronic acid
MSC	mesenchymal stem cell
MMP	matrix metalloproteinase
PEG	poly (ethylene glycol)
PGA	poly (glycolic acid)

## Appendix A

**Bioink**: Any fluid material containing cells that is utilized to fabricate tissue-like products through 3D printing.
**Bioprinting**: Deposition of mixtures of biomaterials and cells, layer by layer, in a bid to recapitulate 3D tissue and organ. Bioprinting is sometimes referred to as an additive manufacturing process.
**Cell-derived extracellular matrix**: The matrix produced after the removal of cells from cell culture system.
**Collagen**: An extracellular matrix component that provides tensile strength to tissues and organs and aids in cellular proliferation and survival.
**Decellularized tissue**: Tissue that has been treated with reagents known to remove cells such as ethanol, sodium hydroxide, and isopropanol.
**Decellularization**: The removal of cells from tissue or organ whilst preserving the ECM components of the tissue or organ.
**Decellularized extracellular matrix**: The remaining microenvironment after removal of cells from tissue and organ. This microenvironment is made up of preserved ECM components in a state similar to that of living tissue or organs.
**Fibronectin**: A glycoprotein that is a major component of the ECM and plays crucial roles in cellular adhesion and migration.
**Gelatin**: Hydrolyzed collagen with numerous free amine groups for crosslinking and gelation.
**Glycosaminoglycans (GAG)**: Unbranched polysaccharides present in ECM and are responsible for many functions including sequestering growth factors.
**Growth factors**: Biomolecules involved in the maintaining and enhancing cellular proliferation, viability, and function.
**Pepsin**: Protease enzyme utilized for acid hydrolysis of gelation.
**Scaffold**: A physical structure that supports cellular functions and interactions.
**Trypsin**: An enzyme used to detach cells from tissue or a surface. Usually used together with EDTA. Naturally found in the digestive system of animals.
